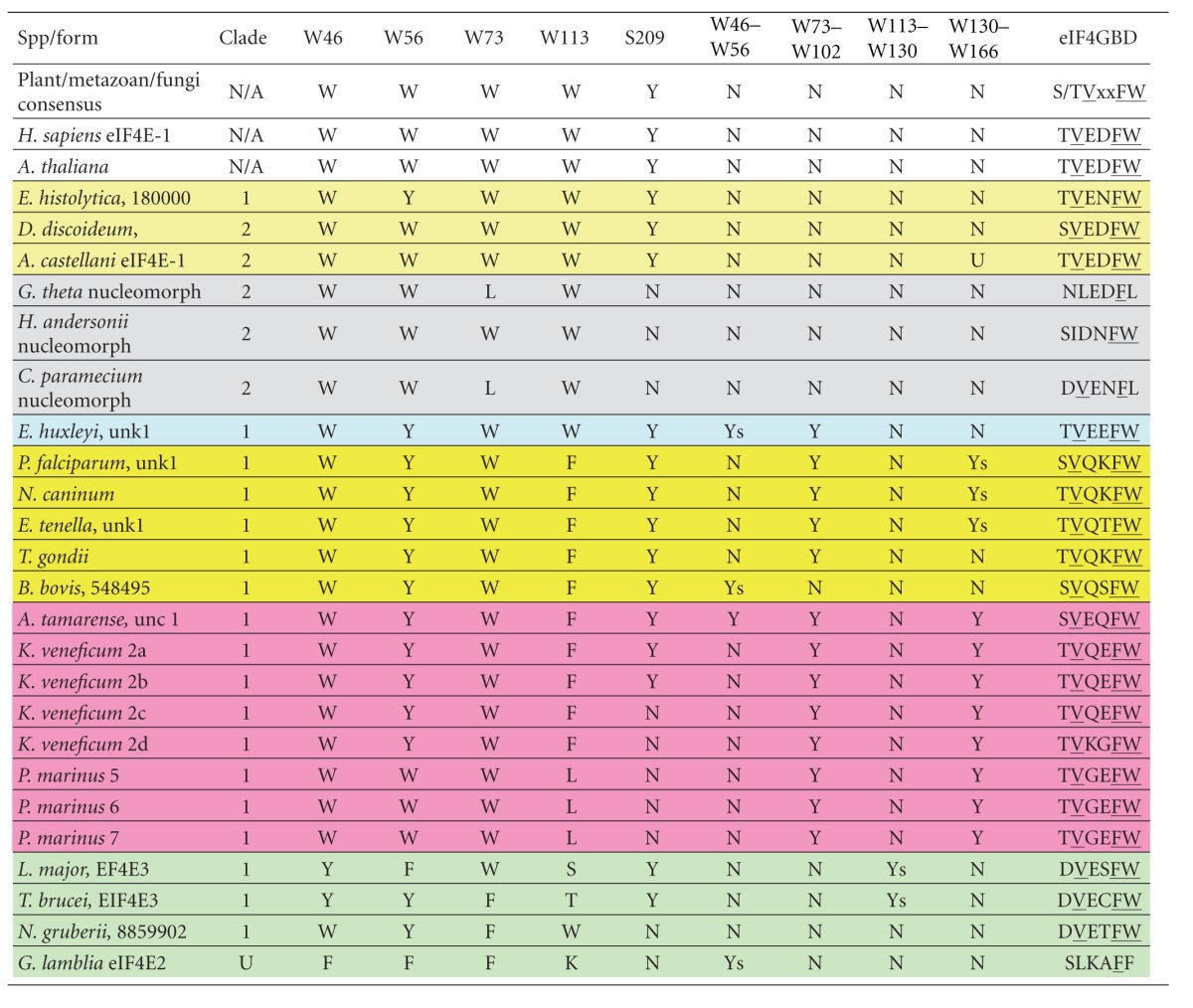# Diversity of Eukaryotic Translational Initiation Factor eIF4E in Protists

**DOI:** 10.1155/2012/134839

**Published:** 2012-06-20

**Authors:** Rosemary Jagus, Tsvetan R. Bachvaroff, Bhavesh Joshi, Allen R. Place

**Affiliations:** ^1^Institute of Marine and Environmental Technology, University of Maryland Center for Environmental Science, 701 E. Pratt Street, Baltimore, MD 21202, USA; ^2^Smithsonian Environmental Research Center, 647 Contees Wharf Road, Edgewater, MD 21037, USA; ^3^BridgePath Scientific, 4841 International Boulevard, Suite 105, Frederick, MD 21703, USA

## Abstract

The greatest diversity of eukaryotic species is within the microbial eukaryotes, the protists, with plants and fungi/metazoa representing just two of the estimated seventy five lineages of eukaryotes. Protists are a diverse group characterized by unusual genome features and a wide range of genome sizes from 8.2 Mb in the apicomplexan parasite *Babesia bovis* to 112,000-220,050 Mb in the dinoflagellate *Prorocentrum micans*. Protists possess numerous cellular, molecular and biochemical traits not observed in “text-book” model organisms. These features challenge some of the concepts and assumptions about the regulation of gene expression in eukaryotes. Like multicellular eukaryotes, many protists encode multiple eIF4Es, but few functional studies have been undertaken except in parasitic species. An earlier phylogenetic analysis of protist eIF4Es indicated that they cannot be grouped within the three classes that describe eIF4E family members from multicellular organisms. Many more protist sequences are now available from which three clades can be recognized that are distinct from the plant/fungi/metazoan classes. Understanding of the protist eIF4Es will be facilitated as more sequences become available particularly for the under-represented opisthokonts and amoebozoa. Similarly, a better understanding of eIF4Es within each clade will develop as more functional studies of protist eIF4Es are completed.

## 1. Eukaryogenesis and Protein Synthesis

Protein synthesis is an ancient, conserved, complex multienzyme system, involving the participation of hundreds of macromolecules in which the mRNA template is decoded into a protein sequence on the ribosome. The ribosome, a complex and dynamic nucleoprotein machine, provides the platform for amino acid polymerization in all organisms [1, 2]. This process utilizes mRNAs, aminoacyl tRNAs, and a range of protein factors, as well as the inherent peptidyl-transferase activity of the ribosome itself. The common origin of protein synthesis in all domains of life is evident in the conservation of tRNA and ribosome structure, as well as some of the additional protein factors. Although the basic molecular mechanisms are conserved across the three domains of life, the Bacteria (eubacteria), Archaea (archaebacteria), and Eukarya (eukaryotes), important divergences have taken place as eukaryotic species have evolved. The origin of the eukaryotic cell is enigmatic. Eukaryotes are thought to have evolved from a fusion of a euryarchaeon with a deep-rooted Gram-positive proteobacteria, the phylum from which mitochondria are derived [3]. It is currently unclear whether the eubacterial fusion partner was distinct from the ancestor of mitochondria or identical to it. This view of the origins of eukaryotes is consistent with the observation that informational genes such as those involved in transcription, translation, and other related processes are most closely related to archaeal genes, whereas operational genes such as those involved in cellular metabolic processes including amino acid biosynthesis, cell envelope, and lipid synthesis are most closely related to eubacterial genes [4]. Such an origin is also consistent with the eukaryotic rooting implied by the presence of an insert within the elongation factor EF-1A that is found in all known eukaryotic and eocytic (crenarchaeal) EF-1A sequences, but lacking in all paralogous EF-G sequences [3].

The mechanisms underlying protein synthesis in all organisms share common features and can be divided into three stages: *initiation*, *elongation*, *and termination*. During *initiation*, the ribosome is assembled at the initiation codon in the mRNA with a methionyl initiator tRNA bound in the peptidyl (P) site. During *elongation*, aminoacyl tRNAs enter the acceptor (A) site and the ribosome catalyzes the formation of a peptide bond. After the tRNAs and mRNA are translocated bringing the next codon into the A-site, the elongation process is repeated until a stop codon is encountered. During *termination*, the completed polypeptide is released from the ribosome, after which the ribosomal subunits are dissociated and the mRNA released for reuse. Different sets of protein accessory factors, the translation factors, assist the ribosome at each of these stages. These are referred to as initiation factors, elongation factors, and termination factors, respectively, to reflect the stage at which they are involved. The elongation process and machinery is well conserved from bacteria to eukaryotes, as is termination. However, the mechanisms of the initiation process, including recognition of the correct reading frame, differ, as do the mechanisms by which mRNA is recruited by the ribosome. Genomewide sequencing projects now allow us to assess the components of translational initiation in a wide range of organisms [5, 6].

Our view of protein synthesis is based mainly on information derived from *S. cerevisiae*, *Drosophila*, plant, and mammalian systems, with the translation components identified through sequencing projects. However, these are only narrow windows on the full diversity of extant eukaryotes. The greatest diversity of eukaryotic species is to be found within the protists, with plants and metazoans representing just two of the estimated 75 lineages of eukaryotes [7, 8]. We are only just beginning to uncover the vast diversity of bacterial-sized (pico- and nano-) eukaryotes, first discovered in clone libraries derived by PCR amplification of pooled “environmental” DNAs (culture-independent PCR) [9–11]. Microbial eukaryotes are a diverse group of organisms characterized by many unusual genome features. These features challenge some of the concepts and assumptions about regulation of gene expression in eukaryotes. In this paper, we will focus on a comparison of our current knowledge of the translation initiation factor eIF4E and its family members from protists. We will compare eIF4E in a range of protists and look at translational components in a simplified translation system found in an algal endosymbiont.

The control of gene expression is a complex process. Even after mRNA is transcribed from DNA, mRNAs can undergo many processing and regulatory steps that influence their expression [12]. Gene regulation at the translational level is widespread and significant. The extent of gene regulation at the translational level has been demonstrated during early *Drosophila* embryogenesis on a genomewide basis that was investigated by determining ribosomal density and ribosomal occupancy of over 10,000 transcripts during the first ten hours after egg laying in *Drosophila*. The diversity of the translation profiles indicates multiple mechanisms modulating transcript-specific translation with cluster analyses suggesting that the genes involved in some biological processes are coregulated at the translational level at certain developmental stages [13]. Similarly, protists have been shown to regulate translation over wide range of conditions and physiological changes, with groups like the dinoflagellates showing regulation of translation to be the predominant form of regulation of gene expression.

## 2. Origin of Eukaryotes

Eubacteria and Archaea show tremendous diversity in their metabolic capabilities but have limited morphological and behavioral diversity; conversely, eukaryotes share similar metabolic machinery but have tremendous morphological and behavioral diversity. Eukaryotes are thought to have evolved from the endosymbiosis of an *α*-proteobacteria and a phagotropic euryarchaeon approximately 2 billion years ago. The transition from prokaryotes to eukaryotes was the most radical change in cell organization since life began, with a burst of gene transfer, duplication, and the appearance of novel cell structures and processes such as the nucleus, the endomembrane system, actin-based cytoskeleton [14, 15], the spliceosome and splicing, nonsense-mediated decay of mRNA (NMD), and ubiquitin signaling [16, 17]. Although the deep phylogeny of eukaryotes currently should be considered unresolved, Koonin and his colleagues have postulated that the mitochondrial endosymbiont spawned an intron invasion which contributed to the emergence of these principal features of the eukaryotic cell [18–20]. Phagocytosis is thought to be central to the origin of the eukaryotic cell for the acquisition of the bacterial endosymbiont that became the ancestor of the mitochondrion. Findings suggest a hypothetical scenario of eukaryogenesis under which the archaeal ancestor of eukaryotes had no cell wall (like modern *Thermoplasma*) but had an actin-based cytoskeleton that allowed the euryarcheon to produce actin-supported membrane protrusions. These protrusions would enable accidental, occasional engulfment of bacteria, one of which would eventually became the mitochondrion. The acquisition of the endosymbiont triggered eukaryogenesis. From a fused cell with two independent prokaryotic gene expression systems, coordination of cell division developed and gene transfer took place through occasional membrane lysis. Some of eubacterial genes recombined into host chromosomes including group II introns [18]. Group II introns can be found among free-living *α*-proteobacteria, the ancestors of mitochondria [21]. They evolved specifically from group II introns that invaded the ancestrally intronless eukaryotic genome through the mitochondrial endosymbiont, thereby generating the prediction that group II introns should be found among free-living-proteobacteria, the ancestors of mitochondria [21]. This prediction was borne out supporting the idea that introns could originate from the mitochondrial endosymbiont. The mobility of group II introns in contemporary eubacteria [22] and their prevalence in *α*-proteobacteria [23] are consistent with such a view. The rapid, coincidental spread of introns following the origin of mitochondria is posited as the selective pressure that forged nucleus-cytosol compartmentalization [18, 20]. The function of the nuclear envelope was to allow mRNA splicing, which is slow, to go to completion so that translation, which is fast, would occur only on mRNA with intact reading frames. The evolutionary relationships of proteins specific to the nuclear envelope and nuclear pore complex reveal that this protein set is a mix of proteins and domains of archaebacterial and eubacterial origins, along with some eukaryotic innovations, suggesting that the nucleus arose in a cell that already contained a mitochondrial endosymbiont [24].

## 3. Evolution of Translational Initiation and Eukaryogenesis

Eukaryotes inherited from their archaeal ancestor a core of translation initiation factors, which includes eukaryotic initiation factor (eIF)1, eIF1A, eIF2 (all three subunits), eIF2B (*α*, *β*, and *δ* subunits only) subunits), eIF4A, eIF5B, and eIF6 [25–27]. The establishment of the nuclear membrane resulted in the physical separation of transcription and translation and presented early eukaryotes with a different challenge; how to shuttle RNA from the nucleus to the site of protein synthesis in the cytoplasm. In prokaryotes, mRNA is translated as it is being synthesized, whereas in eukaryotes, mRNA is synthesized, and processed in the nucleus, and it is then exported to the cytoplasm. There is also a transition from uncapped and polycistronic mRNAs recognized by the ribosome through the Shine-Dalgarno sequence in the 5′-UTR to capped, polyadenylated, and, in most cases, monocistronic mRNAs and the evolution of the scanning process. The evolution of protein synthesis in the context of eukaryogenesis has been discussed previously by Hernández who proposed that recruitment of mRNAs in early eukaryotes was likely to have been through internal ribosome entry sites (IRESs) based on the functional similarity between IRESs and introns [28]. Although not universal, IRES transacting factors (ITAFs) are required for the proper functioning of most viral and cellular IRESs [29, 30]. ITAFs are predominantly nuclear proteins that also play key roles in pre-mRNA splicing and mRNA transport to the cytoplasm [31, 32]. Furthermore, polypyrimidine tracts, a hallmark of introns, are a common feature of cellular and some viral IRESs [33–35]. Hernández considers that the cellular IRESs are descendants of spliceosomal introns and that some of the ITAFs that existed as components of the splicing machinery (such as the ancestral PTB and hnRNPCs) were later incorporated into the nascent eukaryotic translational process. During this period, 5′-UTRs lacking Shine-Dalgarno motifs that were able to passively recruit the 40S ribosomal subunit would have been positively selected and could, therefore, have become the first examples of an IRES [28].

It also seems possible that capped spliced leader (SL) *trans*-spliced mRNAs may have arisen with eukaryogenesis and represent an early form of 5′ blocked mRNAs. In *trans*-splicing, a short SL exon is spliced from a capped small nuclear RNA and is transferred to pre-mRNA, thereby becoming the 5′-terminal end. The fully functional spliceosome is likely to have existed in the last eukaryote common ancestor, leading to splicing components and pre-mRNA signals that are found throughout eukaryotes and are similar among different eukaryotic lineages. It seems certain that SL *trans*-splicing arose through evolution from *cis*-splicing or *vice versa*. *Trans-*splicing shares the splicing signals and most of the components with *cis*-splicing, indicating a common relationship (reviewed [36]). Considering the similarities between the SL snRNP and the spliceosomal snRNPs, specialized *trans*-splicing SL RNAs could have arisen from a splicing U snRNP in ancestral *cis*-splicing early eukaryote and thus may be an ancient form of 5′-end blocking for emerging eukaryotes. SL *trans*-splicing is now found sporadically across the eukaryotic tree of life in a set of distantly related animal groups including urochordates, nematodes, flatworms, and hydra, as well as in the protist Euglenozoa and dinoflagellates, stimulating the argument that a common evolutionary origin seems unlikely. However, an attractive hypothesis to explain multiple evolutionary origins for the SL genes is that they have derived repeatedly from U-rich small nuclear RNAs (snRNAs) of the Sm-class involved in the nuclear spliceosome machinery [37]. In support of this, phylogenomic studies from *Hydra* indicate that SL genes can evolve rapidly in any organism because constraint on SL exon sequence evolution is low [38]. Furthermore, it has been reported that mammalian cells, which do not have SL *trans*-splicing, can SL *trans*-splice when supplied with the SL RNA of either nematodes or trypanosomes [39]. Duplications of the U1 snRNA gene followed by just a few mutations would be sufficient to lead to the acquisition of *trans*-splicing [39] suggesting that it could have happened in the emerging eukaryote as well as in more recent eukaryotic lines.

The separation of the nucleus from the cytoplasm led to the need for mechanisms to shuttle the transcripts into the cytoplasm and to provide for their protection against degradation. With the exception of eIF5, all the eukaryotic-specific initiation factors that evolved, eIF4E, eIF4G, eIF4B, eIF4H, and eIF3, are involved in the 5′-cap-binding and scanning processes. The 5′-cap structure provides stability from 5′ exonucleases and in extant eukaryotes is recognized by the small ribosomal subunit through the novel eukaryotic initiation factor eIF4E. eIF4E, a translational initiation factor found only in eukaryotes, has a unique alpha/beta fold that is considered to have no homologues outside the eukaryotes, as determined by sequence comparison or structural analyses [25]. Although in extant eukaryotes the main role of eIF4E is in translational initiation through cap recognition, it is possible that the cap structure and eIF4E emerged among the primary adaptive responses to the intron invasion and the need for nucleocytoplasmic RNA export, but initially had no role in translation [40]. For instance, it could have appeared in early eukaryotes either as a mediator of nuclear export of mRNAs, thus enhancing mRNA stability during nuclear export, or as a mediator of cytoplasmic storage of mRNAs. Consistent with this, one of the eIF4E proteins from the primitive eukaryote species *Giardia lamblia* binds only to nuclear noncoding small RNAs and has no function in translation [41]. eIF4E is found within different cytoplasmic bodies involved in such processes as mRNP remodeling, mRNA decay or storage [42–44]. In addition, a fraction of this protein resides in the nucleus where it mediates the export of specific mRNAs to the cytoplasm [44, 45]. Since eIF4E has no ability to interact directly with the ribosome itself, the recruitment of eIF4E-bound mRNAs in emerging eukaryotes was likely to have been IRES-dependent.

## 4. Diversity of eIF4E Family Members

In eukaryotes, eIF4E is a central component in the initiation and regulation of translation in eukaryotic cells [46–49]. Through its interaction with the 5′-cap structure of mRNA and its translation partner, eIF4G, eIF4E functions to recruit mRNAs to the ribosome [46]. The interaction of eIF4E and eIF4G can be competed out by a family of 4E-binding proteins, the 4E-BPs, which are capable of repressing translation [46]. Three-dimensional structures of eIF4Es bound to cap-analogues resemble “cupped-hands” in which the cap-structure is sandwiched between two conserved Trp residues (W56 and W102 of *H. sapiens* eIF4E) [50–52]. A third conserved Trp residue (W166 of *H. sapiens* eIF4E) recognizes the 7-methyl moiety of the cap-structure. Aromatic residues Trp, Phe, and His show a distinctive pattern across from N- to C-terminus of the conserved core, containing eight similarly spaced tryptophans summarized by W(x2)W(x8–12)W(x17–20)W(x29–31)W(x9–12)W(x17)W(x32–36)W [6]. Multiple eIF4E family members have been identified in a wide range of organisms that includes plants, flies, mammals, frogs, birds, nematodes, fish, and various protists [53–55]. Evolutionarily, it seems that a single early eIF4E gene underwent a series of gene duplications, generating multiple structural classes and in some cases subclasses. Today, eIF4E and its relatives comprise a family of structurally related proteins within a given organism, although not all function as prototypical initiation factors. Sequence similarity is highest in a core region of 160 to 170 amino acid residues identified by evolutionary conservation and functional analyses [6]. Prototypical eIF4E is considered to be eIF4E-1 of mammals, eIF4E and eIF (iso)4E of plants, and eIF4E of *Saccharomyces cerevisiae*. With the exception of eIF4Es from protists, all eIF4Es can be grouped into one of three classes [6].

Class I members from Viridiplantae, Metazoa, and Fungi carry Trp residues equivalent to W43, W46, W56, W73, W102, W113, W130, and W166 of *H. sapiens* eIF4E-1 [6]. Prototypical eIF4Es bind the cap and eIF4G through the motif S/TVE/DE/DFW in which the Trp is W73. Substitution of a nonaromatic amino acid for W73 has been shown to disrupt the ability of eIF4E to interact with eIF4G and 4E-BPs [56, 57]. Substitution of a Gly residue in place of V69 creates an eIF4E variant that still binds 4E-BP1 but has a reduced capacity to interact with both eIF4G and 4E-BP2 [56]. A serine at residue equivalent to S209 in *H. sapiens* eIF4E-1 is the site of phosphorylation. Only Class I eIF4Es are known to function as translation factors. Genes, and cDNAs encoding members of Class I can be identified in species from plants/metazoans/fungi. As judged from completed genomes, many protists also encode Class I-like family members although these have proven hard to characterize and can show extension or compaction relative to prototypical eIF4E family members [6]. Evidence for gene duplication of Class I eIF4E family members can be found in certain plant species, as well as in nematodes, insects, chordates, and some fungi [53–55]. Class I members include the prototypical initiation factor but may also include eIF4Es that recognize alternative cap structures such as IFE-1, -2, and -5 of *Caenorhabditis elegans* [58, 59], or eIF4Es that fulfill regulatory functions such as the vertebrate eIF4E-1Bs [55, 60–62].

Class II members possess W→Y/F/L and W→Y/F substitutions relative to W43 and W56 of *H. sapiens* eIF4E. These substitions are absent from the model ascomycetes *S. cerevisiae* and *Schizosaccharomyces pombe*. Mammalian eIF4E-2 (Class II) binds only to cap and 4E-BPs [54]. They have been shown to regulate specific mRNA recruitment in *Drosophila* [63] and *C. elegans* [64].

Class III members possess a Trp residue equivalent to W43 of *H. sapiens* eIF4E but carry a W→C/Y substitution relative to *H. sapiens* W56. They have been identified primarily in chordates with rare examples in other Coelomata and in Cnidaria [6, 54]. Their biological function has not yet been determined, although mouse eIF4E-3 has been shown to bind both cap and eIF4G [54]. The protist eIF4Es do not fall into any of these three classes and by plant/metazoan/fungal standards appear to be compacted or possess extended sequences between the conserved tryptophans [6].

## 5. Diversity of Protists and Evolution of Eukaryotic Lineages

The greatest diversity of eukaryotic species is to be found within the protists. Eukaryotes appear to be monophyletic; all extant eukaryotes appear to postdate the acquisition of mitochondria. However, their phylogeny is currently not widely agreed upon. Molecular phylogenetics has the potential to resolve the systematics of eukaryotes. Sequence data continues to accumulate, but with few protists and fewer protist taxa and a distinct bias towards parasites infecting humans (and crop plants). There is increasing availability of multigene data from diverse lineages, although it seems likely that eukaryotic taxonomy will be further complicated by the discovery of ultrasmall eukaryotes. These are scattered across the eukaryotic tree and may include major new supergroups [9, 65]. The root of the eukaryotes remains open to debate, but recent analysis places the eukaryotic root between the monophyletic “unikonts” and “bikonts” [66].

The protists are defined loosely as unicellular eukaryotic organisms that are not plants, animals, or fungi. Eukaryotic features evolved within the protists that thrived for up to a billion years before they gave rise independently to multicellular eukaryotes, the familiar plants, animals, and fungi [67]. Extreme examples of genome sizes, both large and small, can be found among microbial eukaryotes from 8.2 Mb in the apicomplexan *Babesia bovis* to >200,000 Mb in certain dinoflagellates. Roughly forty sequenced genomes are available (depending on classification), some of which are multiple representatives of the same genus, for example, *Plasmodium*, *Leishmania*, and *Trypanosoma*. The last common ancestor of all eukaryotes is believed to have been a phagotrophic protist with a nucleus, at least one centriole and cilium, facultatively aerobic mitochondria, sex (meiosis and syngamy) and a dormant cyst with a cell wall of chitin and/or cellulose, and peroxisomes (based on a root along the lineage leading to Euglenozoa). Endosymbiosis led to the spread of plastids. Analyses of multigene genealogies have led to the conclusion that the acquisition of photosynthesis in eukaryotes arose from a primary endosymbiosis between a cyanobacterium and a eukaryotic host. This gave rise to glaucocystophytes (white lineage), red algae (red lineage), and green algae (green lineage, including plants) [7, 68–70]. Plastids spread by secondary endosymbiosis. Other photosynthetic eukaryotes such as cryptomonads, haptophytes, chlorarachniophytes (amoeboflagellate cercozoans), dinoflagellates, diatoms, brown algae, and euglenids are the result of secondary endosymbiosis, tertiary endosymbiosis, and, perhaps, even quaternary endosymbiosis in which a nonphotosynthetic eukaryotic ancestor engulfed a photosynthetic eukaryote [68, 71, 72]. Endosymbiosis resulted in the transfer of hundreds of genes to the host nucleus. Multiple gains and multiple losses of plastids are likely to have occurred, with plastids possibly lost in ciliates and remaining in relict form in apicomplexans [73] and *Perkinsus* [74]. Dinoflagellates have substituted the ancestral plastid several times by tertiary symbioses involving a diverse array of eukaryotes [71, 72].

**Figure 1:**
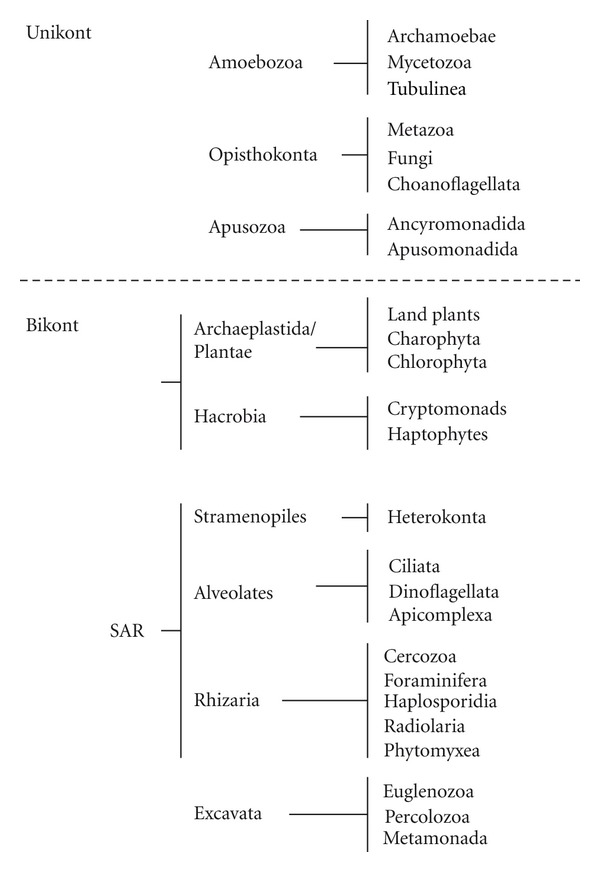
Relationships among major lineages of eukaryotes. Summary tree of eukaryotic relationships based on multigene analyses as outlined by Parfrey et al. [8].

There is no real consensus on eukaryotic phylogeny currently; part of the problem is that we are still very much in the discovery phase, and another is that some of the divisions are quite ancient. In recent years, eukaryotic taxonomy has shifted towards a new system of six supergroups that aims to portray evolutionary relationships between microbial and macrobial lineages [8, 75–77]. The six supergroups posited are the Amoebozoa, Opisthokonta, Apusozoa, the Archaeplastida/Plantae, SAR (Stramenopiles, Alveolates, and Rhizaria), and the Excavata (Table 1). These break down into two larger groups, those with a single flagellum (unikonts), which may or may not be retained, and those with two flagella (bikonts) (Table 1). A summary tree of eukaryotic relationships based on multigene analyses as outlined by Parfrey et al. [8] is shown in Figure 1.

The Amoebozoa includes a diversity of predominantly amoeboid members such as the tubulinid amoeba, *Amoeba* spp., *Dictyostelium discoideum* (cellular slime mold), and *Entamoeba* spp., which are secondarily amitochondriate. Opisthokonts include the metazoans, fungi, and the choanoflagellates such as *Monosiga brevicollis* that are the sister to the metazoans [78]. This is the best supported supergroup. The Apusozoa is a supergroup comprising flagellate protozoa, the apusomonads, and ancyromonads. On molecular trees, these two group together, but their relationship to other eukaryotes is uncertain [8]. The supergroup Archaeplastida/Plantae was posited to unite the three lineages with primary plastids: green algae (including land plants), rhodophytes, and glaucophytes with two other lineages, the cryptophytes and haptophytes, both of which have secondary plastids [79]. There is strong support for the SAR supergroup consisting of stramenopiles, alveolates, and plus rhizarians [8]. Within the SAR clade, each of the three members forms distinct lineages [68, 70]. For example, the Rhizaria emerged from molecular data to unite a heterogeneous group of flagellates and amoebae including cercomonads, foraminifera, diverse testate amoebae, and former members of the radiolaria [80] and represents an expansion of the Cercozoa to include foraminifera [81]. The Cercozoa was also recognized from molecular data [82]. Cercozoa and foraminifera appear to share a unique insertion in ubiquitin [83], although there is a paucity of nonmolecular characters uniting the members of this supergroup [8]. Within the alveolates, the Apicomplexa is a large monophyletic group many of which are parasites, including *Plasmodium*, the parasite responsible for malaria. The last supergroup is the Excavata, a supergroup composed predominately of heterotrophic flagellates, and includes many important parasites such as the trypanosomes, *Giardia*, and trichomonads. Within this supergroup, the “euglenozoa,” the combination of eugleniids and trypanosomes is a grouping with good support.

## 6. Unusual Features of Protist eIF4Es

**Figure 2:**
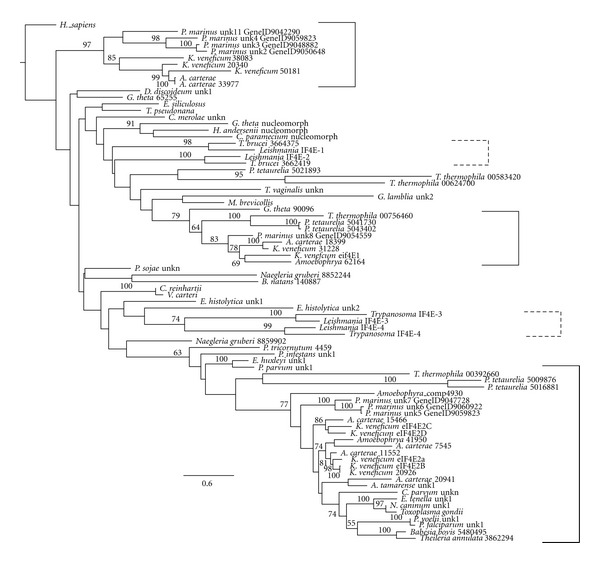
Relationship of selected eIF4E-family members from multiple protist species. Maximum likelihood phylogeny of eIF4E amino acid sequences aligned with T-coffee and trimmed to include only the core region of 453 aligned positions (corresponding to positions 30 to 203 of the human sequence). The tree was constructed using RAxML with the Jones Taylor Thornton gamma distributed model with 100 rapid bootstrap replicates. Bootstrap values above 50% are shown.

A previous phylogenetic analysis of eIF4E family members from protists indicated that they cannot be grouped with the three main classes that describe eIF4E family members from multicellular organisms [6]. At the time of the earlier analysis, very few sequences were available for protists. Many more are now available, though not all in publically available databases.  Figure 2 shows a tree describing the overall relationships of selected eIF4E-family members from multiple protists species rooted with *H. sapiens* eIF4E-1. The tree shows maximum likelihood phylogeny of eIF4E amino acid sequences aligned with T-coffee and trimmed to include only the core regions corresponding to amino acids 30 to 203 of the human sequence). The tree was constructed using RAxML with the Jones Taylor Thornton gamma distributed model with 100 rapid bootstrap replicates. Bootstrap values above 50% are shown. Sequences derive predominantly from representatives of SAR mainly heterokonts, ciliates, apicomplexans, dinoflagellates, *Perkinsus*, along with Excavata representatives from Diplomonads (*Giardia*), Euglenozoa (*Trypanosoma*), and Parabasalids (*Trichomonas*).

Three clades stand out and are bracketed with solid lines. All three solid bracketed clades include eIF4Es from dinoflagellates and *Perkinsus* suggesting the possibility of three different classes. The bottom bracket shows a large clade (Clade 1) including eIF4Es from the ciliate, *Tetrahymena thermophila*; *Perkinsus marinus* eIF4E-5, -6, and -7; eIF4E-2a–d sequences from the dinoflagellate, *Karlodinium veneficum*, along with eIF4Es from the dinoflagellates *Amphidinium carterae *and *Amoebophrya*. This clade also includes eIF4Es from the closely related apicomplexans and is the only strong clade with apicomplexans in this tree. Clade 1 also includes “dotted line” clade eIF4E family members from the euglenozoan excavates, *Leishmania* and *Trypanosoma*, EIF4E3 and 4. The next bracketed clade (Clade 2) includes eIF4E family members from *K. veneficum *(eIF4E-1), *A. carterae *18399, *P. marinus* eIF4E-8, *Amoebophrya* and the ciliate *T. thermophila* and “dotted line” clade that includes trypanosome sequences *Leishmania* EIF1 and 2. Characteristics of some Clade 1 and Clade 2 eIF4E family members are summarized in Table 2. The top bracketed clade (Clade 3) contains eIF4E family members from *P. marinus*, eIF4E-2, -3, -4, -11, *K. veneficum* eIF4E-1, and *A. carterae* 33977. eIF4Es from ciliates are absent from this top clade, and there is an “orphaned” clade of ciliate sequences. These results suggest gene duplication into three groups prior to divergence of the alveolates with the loss of one copy in *Amoebophrya* and the loss of two copies in apicomplexans. An alternate explanation could be that these copies are not apparent because they are so diverged, or, in the case of *Amoebophrya*, because of poor coverage.

## 7. eIF4E Family Members in *Giardia lamblia*

*Giardia lamblia* is an amitochondriate flagellated protozoan parasite that belongs to the diplomonad group (Excavata) that includes both parasitic and free living species [84]. Its genome is compact in structure and content (~11.7 Mb), contains few introns or mitochondrial relics, and has simplified machinery for DNA replication, transcription, RNA processing, and most metabolic pathways [85]. mRNA recruitment in these organisms is unusual in that their transcripts have exceedingly short 5′ untranslated regions (5′-UTRs), ranging from 0 to 14 nucleotides, and similarly short 3′-UTRs of 10 to 30 nucleotides [86]. Extremely short 5′-UTRs are a highly conserved trait of transcripts from *Trichomonas*, *Entamoeba,* as well as *Giardia*. The precise cap structure in *Giardia* RNAs has not yet been determined, although native *Giardia* mRNAs have blocked 5′-ends and the genome encodes a yeast-like capping apparatus [87]. Furthermore, m^7^GpppN-capped mRNA introduced into the cells is expressed well [87, 88]. Eight m^2,2,7^GpppN-capped snRNA species have been identified in *Giardia* [89]. Experimentally, mRNA recruitment occurs efficiently in mRNAs that are capped and in which the first initiation codon is located only 1 nucleotide downstream from the m^7^GpppN-cap structure. Recruitment can be decreased when the 5′-UTR between the cap and the initiation codon is lengthened beyond 9 nucleotides [88]. There are two eIF4E family members in *Giardia*, termed eIF4E1 and eIF4E2, which have distinct properties [41]. Of the two, eIF4E2 has been shown to be essential and binds to m^7^GTP-Sepharose, suggesting that it functions in protein synthesis. The other, eIF4E1, is not essential and binds only to m^2,2,7^GpppN-Sepharose. eIF4E1 is found concentrated and colocalized with the m^2,2,7^GpppN cap, 16S-like rRNA, and fibrillarin in the nucleolus-like structure in the nucleus [41]. Of the eight conserved tryptophan residues typical of eIF4E Class I sequences, both forms have a Phe residue at the position equivalent to human W56. eIF4E1 has Leu at the position equivalent to human W73, and eIF4E2 has a Phe residue (Table 2). Both forms have poor consensus at the eIF4G binding site with substitutions of W113/Y and W113/I for eIF4E1 and eIF4E2, respectively (numbering as in human eIF4E), eIF4E1 has an insertion between residues 130–166. 

## 8. eIF4E Family Members in Trypanosomatids

Trypanosomatids are a group of kinetoplast protozoa (Excavata/Euglenozoa) distinguished by having only a single flagellum. The haploid genome size in *Leishmania major* is ~36 Mb (haploid). mRNA maturation in trypanosomes differs from the process in most eukaryotes mainly because protein-coding genes are transcribed into polycistronic RNAs in this organism [36, 37, 90]. Transcription of protein coding genes occurs polycistronically, and processing to monocistronic mRNAs occurs through coupled splice leader (SL) *trans*-splicing and polyadenylation (reviewed [36]). The SL *trans*-splicing mechanism was once considered an anomaly of the kinetoplastids, but subsequent identification of *trans*-splicing in dinoflagellates, *Perkinsus*, euglenozoans, and several major invertebrate phyla suggests that this particular form of RNA processing may represent an evolutionarily important aspect of gene expression [36, 37, 91]. There are similarities, particularly in genomic arrangement of SL RNAs, between phyla known to exhibit *trans*-splicing and their mRNAs; however, there is little sequence similarity between the SLs of different organisms. In this RNA-mediated form of *trans*-splicing, a short SL exon is spliced from a capped small nuclear RNA and is transferred to pre-mRNA, thereby becoming the 5′-terminal end and providing an unusual cap structure to mature mRNAs. In *Euglena* (Excavata/Euglenozoa), the SL contribution results in trimethylguanosine, a so-called trimethyl cap, m^2,2,7^GpppG (TMG), in which there are additional methylations to the prototypical monomethyl (m^7^GpppN) cap structure found on most eukaryotic mRNAs [92]. In metazoans such as nematodes, where only a percentage of mRNAs are *trans*-spliced, the SL contribution results in a trimethyl cap [93]. In kinetoplastids, all of the mRNAs are *trans*-spliced and the SL contribution results in a highly unique cap structure where additional methylations are apparent. Whereas no more than three modified nucleotides have been described in any metazoan cap structure, the kinetoplastid cap has four consecutive modifiednucleotides (and thus by convention is referred to as a cap-4 structure) [94, 95]. This has been the most highly modified eukaryotic mRNA cap known to date. In trypanosomatids, mRNAs have a common 39-nt long spliced leader sequence at the distal end of the 5′-UTR, which is identical for all mRNAs of a given species. Regulation of gene expression in trypanosomatids is accomplished mainly through posttranscriptional mechanisms such as control of mRNA stability and translation [96–98].

Four eIF4E family members have been characterized from the trypanosomatids *Leishmania major* and *Trypanosoma brucei*, termed EIF4E1, 2, 3, and 4 [99, 100]. All four are expressed in both procyclic and bloodstream forms of the parasites. These four can be broadly classified into two groups (Figure 2). Sequence analysis has identified features that distinguish EIF4E1 and 2 from EIF4E3 and 4 in both *T. brucei* and *L. major*. Similarly, separation of the four eIF4Es into two distinct groups can be made on the basis of localization and function [100]. In *T. brucei, *EIF4E1 and 2 (Group 1, expanded Clade 2) localize both to the nucleus and the cytoplasm and do not seem to be directly involved in translation based on knockdown experiments, although they do perform functions essential for cellular viability [100]. The second group (Group 2, Clade 1) formed by EIF4E3 and 4 is more abundant, is strictly cytoplasmic, is required for translation, and interacts with *T. brucei *eIF4Gs [100].

Group 1 comprises the EIF4E1 and 2 sequences (expanded Clade 2), which are more similar in size to the human and yeast sequences, but show extensions between W102–W113. The function of this extension in Clade 2 eIF4Es in euglenozoans is not known, but the prolines suggest it is solvent exposed and thus could be involved in protein-protein interaction. eIF4E family members from Group 2 (expanded Clade 1), EIF4E3 and 4, share a few unusual features absent from the Group 1 members and distinct from plant, fungi, and metazoan eIF4Es. These include a long N-terminus of more than 150 amino acids which share extensive homology between different orthologues in the EIF4E3 sequences and also contain short segments of limited homology which seem to be conserved between the EIF4E3 and EIF4E4 sequences [100]. Of the eight conserved tryptophan residues typical of eIF4E Class I sequences, most are either conserved in the various trypanosomatid homologues or are replaced by other aromatic residues such as W56Y/F in the Group 2 eIF4Es (human eIF4E numbering) (Table 2) [100]. The only exception is W113, present in the EIF4E1 and EIF4E2 sequences but which is replaced by nonaromatic hydrophilic residues in EIF4E3 and 4. Other substitutions in the trypanosomatid sequences are D104, next to the universally conserved W102/E103, involved in cap binding [50] which is replaced by a histidine in EIF4E2 and 3; V69/E70, part of the eIF4G-binding domain [101], which is missing in EIF42 and EIF4E4 [100].

EIF4E3, the most abundant *Trypanosoma* and *Leishmania* eIF4E family member, is the only confirmed essential homologue in procyclic and bloodstream T*. brucei*. The similarities observed between *T. brucei* EIF4E3 and 4 at the sequence level, their similar subcellular localization, abundance, and their ability to bind to eIF4G partners are consistent with both performing related-roles in translational initiation. Interestingly, *T. brucei *EIF4E1, 2, and 4, but not *T. brucei *EIF4E3, can efficiently bind the m^7^G cap. Nevertheless, when compared with EIF4E4 in *L. major*, it binds less efficiently to the trypanosomatid cap4 [99]. Although *T. brucei* EIF4E2 binds to the m^7^G Sepharose in a similar manner to *T. brucei* EIF4E1 and 4, *L. major *EIF4E2 does not bind this cap [102], but rather, preferentially binds the methylated cap4 [99]. This difference, plus the existence of unusual insertions in the *L. major* EIF4E2 between W113–W130 that are missing from the *T. brucei* or *T. cruzi* orthologues, implies a divergence in function unique to the *L. major* protein. The earlier prediction [99] that this insertion might be related to the ability of *L. major *eIF4E to bind to the larger cap-4 seems therefore not to be a compelling argument.

## 9. eIF4E Family Members in Dinoflagellates

Dinoflagellates are alveolate unicellular protists and a sister group to the parasitic apicomplexans such as *Toxoplasma gondii* and *Plasmodium falciparum*. Dinoflagellates are a diversified group that exhibit a wide diversity in size, form, and lifestyle. They also show a wide spread of genome size, from 1500 to 4700 Mb in *Symbiodinium *sp to 112,000 to 220,050 Mb in *Prorocentrum micans* [103]. Ninety percent of all dinoflagellates are marine plankton with the remaining species being benthic, freshwater, or parasitic.

The free-living species are major primary producers, and several are known to produce harmful algal blooms that result in massive fish kills, human and marine mammal intoxications, as well as economic losses in fisheries and tourism. However, scientific interest with dinoflagellates extends beyond their ecological and economic importance. They possess numerous cellular, molecular, and biochemical traits not observed in “text-book” model organisms. It appears that the organization and regulation of genes in dinoflagellates is different from that of typical eukaryotes. DNA is in permanently condensed chromosomes not packaged in nucleosomes and DNA content ranging from 3 to 250 pg per cell (up to almost 60-fold larger than humans) [104]. Within the dinoflagellate genome, there appears to be a high degree of DNA redundancy, with multiple tandem copies (>20 in many cases) of protein coding genes to give complex gene families [103, 105] that are highly and coordinately expressed. Unlike trypanosomes, in which polycistronic mRNAs contain a series of different genes, the examples studied in dinoflagellates consist of tandemly arrayed copies of the same gene [105].

Recent studies find a predominance of posttranscriptional control of gene expression in dinoflagellate gene expression, including circadian controlled processes such as bioluminescence [106], carbohydrate metabolism [107], and the cell cycle [108], as well as a range of stressors [109–114]. The Van Dolah lab, at the NOAA Center for Coastal Environmental Health and Biomolecular Research, has developed an oligonucleotide microarray from 11,937 unique ESTs from the dinoflagellate *Karenia brevis* [115]. Following validation of the microarray, large-scale transcript profiling studies were performed examining diurnally regulated genes and genes involved in the acute stress response. These studies represent the largest transcript profiling experiments in a dinoflagellate species to date and showed only a small percentage of transcripts changing. None of the anticipated genes, under transcriptional control in other eukaryotes (e.g., cell cycle genes, heat shock, etc.), showed changes in mRNA abundance. Consistent with this, a massively parallel signature sequencing (MPSS) analysis of the transcriptome of the dinoflagellate *Alexandrium tamarense* has shown that of a total of 40,029, only 18, 2, and 12 signatures were found exclusively in the nutrient-replete, nitrogen-depleted, and phosphate-depleted cultures, respectively. The presence of bacteria had the most significant impact on the transcriptome, although the changes represented only ~1.0% of the total number of transcribed genes and a total of only ~1.3% signatures were transcriptionally regulated under any condition [116]. Since the levels of many proteins have been well documented to change in a variety of dinoflagellates, these large-scale studies point to translational regulation as a likely regulatory point in dinoflagellate gene expression. Currently, almost nothing is known about translational initiation or its regulation in these organisms.

Dinoflagellates have mRNAs with unique spliced leaders and cap structures: through analysis of sequences representing all major orders of dinoflagellates, nuclear mRNAs from fifteen species were recently found to be *trans*-spliced with the addition of a 22-nt conserved SL [117, 118]. SL *trans*-splicing has not been identified in a ciliate or apicomplexan to date; however, preliminary analysis using the 22-nt dinoflagellate SL revealed the usage of *trans*-splicing in *Perkinsus marinus* and *P. chesapeaki*, phylogenetic intermediates between apicomplexans and dinoflagellates [118]. Recently, SL *trans*-splicing has been identified in *Amoebophrya* sp, a member of the *Syndinales*, a dinoflagellate parasite of dinoflagellates, which represents a basal root of the dinoflagellates [119]. This suggests the SL machinery was present in an early ancestor of dinoflagellates. It is unclear whether all or only a subset of dinoflagellate genes are subject to SL *trans*-splicing, but, given the diversity of the cDNAs found in the full length libraries, a conservative estimate would be that greater than 90% of mRNAs are *trans*-spliced.

The 22-nt sequence found in dinoflagellate SL-RNA is 5′A(T)CCGTAGCCATTTTGGCTCAAG-3′ [118]. The identity of the cap structure for the SL-RNA needs to be verified, but preliminary analysis indicates only a monomethylated 5′ m^7^G is present on mRNAs. Based on the SL-RNA sequence and LC-MS analysis, Place has proposed the following novel cap-4 structure for dinoflagellate mRNAs: m^7^GpppA(U)p^m2'^Cp^m2'^CpG with modifications to A (U) and G still needing to be established (unpublished results). There is no evidence for a trimethylguanosine or 2′O-methyl adenosine.

**Figure 3:**
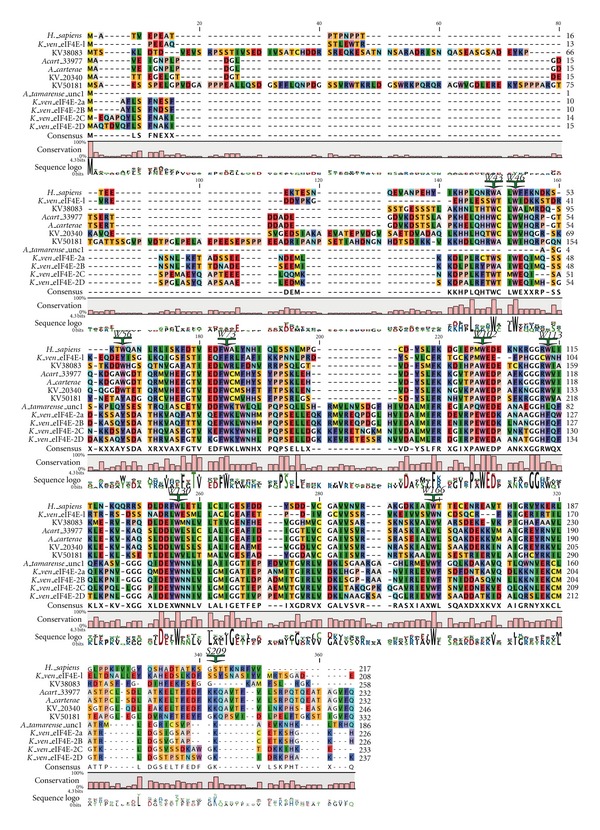
Comparison of the sequences of selected eIF4E-family members from *Karlodinium veneficum* and other dinoflagellates. Alignment of the amino acid sequences of selected established eIF4E-family members from *K. veneficum* and other dinoflagellates. Amino acid sequences were aligned with T-coffee using the BLOSUM62MT scoring matrix in CLC Main Workbench. To facilitate comparison of the sequences, the residues conserved in Class I eIF4Es in multicellular organisms are indicated and numbered as in human eIF4E-1: W43, W46, W56, W73, W102, W113, W130, W166, and S209.

Dinoflagellates encode unusual eIF4E-family members. Two distinct eIF4E orthologues, eIF4E-1 and -2 have been partially characterized in *K. veneficum *(Jagus and Place, m/s in preparation) (Figure 3). To facilitate comparison of the sequences, the residues conserved in Class I eIF4Es in multicellular organisms are indicated and numbered as in human eIF4E-1: W43, W46, W56, W73, W102, W113, W130, W166 and S209. eIF4E-2 is represented by four distinct but closely related subtypes (eIF4E-2a–d) (Figure 3). Seven contigs encoding eIF4E-1 and 31 contigs encoding eIF4E-2 (approximately equivalent representation by the a–d subtypes) have been identified indicating the eIF4E-2 group is more highly expressed and may represent the dominant isoforms in the cell. A neighbor-joining tree predicts that the dinoflagellate eIF4E-2 is related to eIF4Es from the kinetoplasts *Leishmania* and *Trypanosoma* with 51% bootstrap support. RT-qPCR analysis for eIF4E transcript abundance is consistent with this assertion (Jagus and Place, m/s in preparation). The *K. veneficum* eIF4E sequences are aligned in Figure 3 with prototypical eIF4E-1 from human. Also included are the sequences for additional, as yet uncharacterized eIF4E family members. Additional sequences were uncovered after this paper was initiated and are shown as kv20926 and kv31228 in Figure 2; however, their sequences are not included in Figure 3. Kv20926 groups with *K. veneficum* Clade 1 eIF4E-2 subtypes and kv31228 with *K. veneficum* Clade 2 eIF4E-1. *K. veneficum* eIF4Es show a clear separation into two subclasses, based on an insert of 11 amino acids between W73 and W102 (numbering equivalent to human eIF4E-1) and distribute between three clades. *K. veneficum* eIF4E-1 and eIF4E 2a–d have a Tyr substitution at the position equivalent to human W56, one of the tryptophans involved in cap binding. This is also observed in eIF4Es from the dinoflagellate *Alexandrium tamarense*, but not from *Amphidinium carterae*. In addition, eIF4E-1 has glutamine instead of D/E in the eIFG/4E-BP-binding domain. The eIF4E-2 family members contain extended amino acid stretches between the structural units of the core, between residues equivalent to human W73 to W102, and W130 to W166. In addition, eIF4Es from several alveolate species have a Trp to Phe substitution at W113 [6], a characteristic shared by *K. veneficum* eIF4E-1. It is of interest that the different subtypes of *K. veneficum* eIF4E-2s show marked heterogeneity between W102–W113. The conserved phosphorylation site of eIF4E is only observed in eIF4E-2a and -2b of *K. veneficum*. eIF4E-2a and -2b share the TKS motif at the putative phosphorylation site in which the Lys residue is a sumoylation site in human eIF4E [120, 121]. The sumoylation site at the equivalent of human Lys35 is shared by eIF4E-2b, -2b, -2c, and -2d. eIF4E-1 contains the sumoylation site equivalent to human Lys210. eIF4E-2a, but not eIF4E-1, binds to m^7^GTP-Sepharose *in vitro*, although neither interact with TMG. It is not known whether either form interacts with the unique cap-4 of dinoflagellates (Jagus/Place, m/s in preparation). These results are consistent with eIF4E-2a being a functional initiation factor, but not definitive. The *K. veneficum* eIF4E-2s fall into Clade 1 raising the possibility that other eIF4Es of Clade 1 bind to m^7^GTP. The eIF4E-1s fall into Clade 2. Unlike *K. veneficum* eIF4E-1, some of the extended Clade 2 members like the* L. major* and *T. bruceii* eIF4E1 and 2 are known to bind m^7^GTP but appear not to participate in protein synthesis [100], making it hard to predict function of the *K. veneficum* eIF4E-1s. Three of the *K. veneficum *eIF4Es fall into Clade 3. As with the *K. veneficum* Clade 2 representatives, these do not have the insert between W73 and W102.

## 10. eIF4E Family Members in *Perkinsus* *marinus*

**Figure 4:**
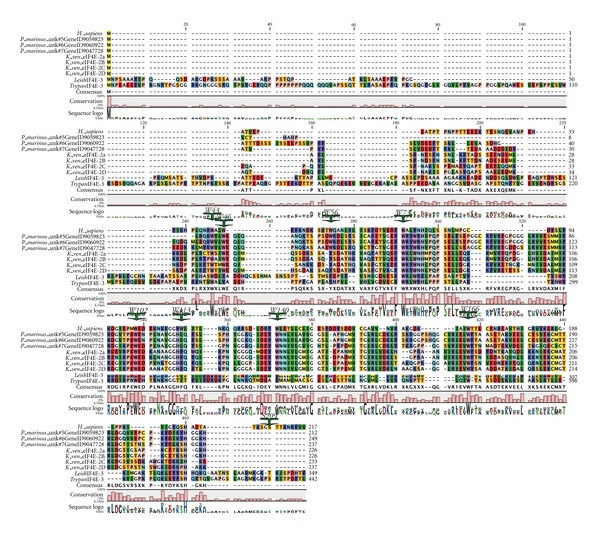
Comparison of the sequences of selected eIF4E-family members from *Perkinsus marinus* with related species from *K. veneficum *and trypanosome species. Alignment of the amino acid sequences of selected established eIF4E-family members from *P. marinus* with related species from *K. veneficum*. Amino acid sequences were aligned with T-coffee using the BLOSUM62MT scoring matrix in CLC Main Workbench. To facilitate comparison of the sequences, the residues conserved in Class I eIF4Es in multicellular organisms are indicated and numbered as in human eIF4E-1: W43, W46, W56, W73, W102, W113, W130, W166, and S209.

*Perkinsus marinus* is an alveolate with a genome of 86 Mb and is closely related to the dinoflagellates [122]. Like the dinoflagellates, it also exhibits *trans*-splicing. Five different SLs of 21-22 nucleotides (nt) in length have been reported from *P. marinus* [123–125]. Variability at positions 1 and 2 between the different SLs suggests variability of cap structures. Overall these data suggest a complex gene regulatory system both at the level of mRNA generation and of translational control consistent with its complex life style. The *P. marinus *genome encodes eight eIF4E family members along with two very large (>600 amino acid) forms that contain only some of the typical eIF4E signatures. *P. marinus* eIF4E-5, -6, and -7 form a group that aligns most closely with the *K. veneficum* eIF4E-2s in Clade 1, suggesting they will bind m^7^GTP caps (Figures 1 and 4 and Table 2). These share the insertions between W73 to W102 and W113 to W133. This group also has TVGEFW at the eIF4G binding domain. In addition, they each have a Trp to Leu substitution at W113. The* L. major* and *T. bruceii* also show a consistent substitution at this position, but to a hydrophilic amino acid. *P. marinus *eIF4E-2, -3, and -4 also form a group in Clade 3 with eIF4Es with two of the *K. veneficum* eIF4Es (Figure 2, Table 2). *P. marinus *eIF4E-8 groups with *K. veneficum* eIF4E-1.

## 11. Cryptomonads, *Guillardia theta*, and Nucleomorphs

**Figure 5:**
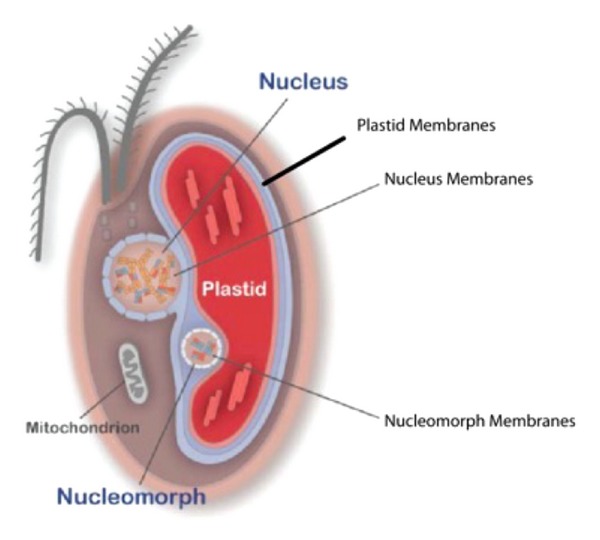
Cellular organization of *Guillardia theta*. Former chloroplast genes now inserted in nucleomorph or nuclear chromosomes are indicated in green, and former red algal genes now in the host nucleus are indicated in red. The four endosymbiont membranes are clearly represented.

Cryptomonads (Chromalveolata/Cryptophyta) are chimeras of two different eukaryotic cells; a flagellate host and a photosynthetic endosymbiont. These organisms are thought to have arisen by secondary symbiogenesis shortly after the origin of the common ancestor of green plants, red, and glaucophyte algae [126–128]. In the cryptomonad *Guillardia theta*, the flagellate host acquired a chloroplast by engulfing and retaining a red alga. In doing so, the host was able to convert from obligate heterotrophy to an autotrophic way of life [129–131]. In addition to the red algal chloroplast, cryptomonads have retained a vestigial red algal nuclear genome as a minute nucleomorph with three chromosomes [132–134]. The nucleomorph resides in a cell compartment, the periplastid space, that also contains the chloroplast. The cellular organization of *Guillardia theta* is shown in Figure 5.

In the cartoon, former chloroplast genes now inserted in nucleomorph or nuclear chromosomes are indicated in green, and former red algal genes now in the host nucleus are indicated in red. The nucleomorph genome has been sequenced and shown to be 551 kbp with a gene density of 1 gene per 977 bp, encoding 464 putative protein coding genes [133]. This compact genome has infrequent overlapping genes, and short inverted repeats containing rRNA cistrons at its chromosome ends [132, 133, 135]. There is almost a total absence of spliceosomal introns which has facilitated gene annotation. Marked evolutionary compaction [126–128] has eliminated almost all the nucleomorph genes for metabolic functions, but left a few hundred housekeeping genes, and 30 genes encoding chloroplast-located proteins [133]. The housekeeping genes are limited to nuclear maintenance and transport, translation, protein degradation and folding, and microtubule/centrosome functions [133, 135]. More than 20% of the housekeeping genes encode components of the translational machinery. The nucleomorph and its periplastid space can be viewed as providing a minimum eukaryotic expression system for a small number of nucleomorph-encoded chloroplast proteins. The endosymbiont has been reduced to an organelle, equivalent to a “complex plastid.” The relict, enslaved red alga is referred to here as the endosymbiont for convenience, although strictly speaking it should be considered an organelle.

## 12. The Translation Machinery of the *Guillardia theta *Nucleomorph

The endosymbiont encodes its own rRNA and 65 ribosomal proteins [133]. Functioning endosymbiont ribosomes have been demonstrated in the endosymbiont cytoplasm [136]. The endosymbiont has its own mRNAs with 5′-caps and poly(A) tails, elongation, and release factors, but only a subset of translational initiation factors. The nucleomorph encodes eIF1, eIF1A, eIF4A, eIF2 (all subunits, although the alpha subunit is truncated), eIF4E (truncated), eIF5B, eIF6, and poly(A) binding protein. It does not appear to encode any of the subunits of eIF2B, the factor that promotes guanine nucleotide exchange on eIF2. Furthermore, several initiation factors thought to be essential for eukaryotic initiation have not been identified; the nucleomorph does not encode eIF4B, eIF5, or the scaffold proteins eIF3 (any subunit) or eIF4G. All of these initiation factors have been shown to be essential in yeast (reviewed [137]). The nucleomorph is also without the eIF4E regulatory proteins, the 4E-BPs. Since the genome of the *G. theta* nucleomorph has been so severely compacted, it is hypothesized that the genes encoding complex cellular functions, such as protein synthesis, are limited to the minimal set needed to accomplish the function. Beyond the reduction in the number of initiation factors, several of the translational initiation factors encoded are truncated compared to their counterparts in nonprotist eukaryotes. This system can be considered to represent a natural experiment in deletion analysis and may tell us much about structure/function relationships in initiation factors, in addition to deepening our knowledge of this branch of the eukaryotic Tree of Life.

The factors eIF1, eIF1A, eIF2, eIF2B, eIF3, eIF4A, eIF4E, eIF4G, and eIF5 are all essential in yeast. eIF5B is not essential, although its deletion produces a severe slow growth phenotype [138]. The possibility that the lack of eIF2B, eIF3, eIF4G, and eIF5 in the *G. theta* endosymbiont reflects a primitive condition is unlikely since the deeply rooted, free-living red alga, *Cyanidioschyzon merolae*, encodes eIF4G, eIF5, and all the subunits of eIF2B and eIF3 [139]. *C. merolae* is considered to have the smallest genome of any free-living photosynthetic organism and molecular analyses support the primitiveness of this alga [140]. However, like the *Guillardia theta* nucleomorph, *C. merolae* does not appear to encode eIF4B, suggesting that eIF4B is a later evolutionary development [139]. Consistent with this, eIF4B is not essential in yeast, although its disruption results in a slow growth and cold-sensitive phenotype [141]. The endosymbiont has either evolved a minimal system of initiation through compaction of the genome, has made mechanistic adjustments to overcome factor deficiencies, or uses host factors. Use of host factors would require transport across the outer two membranes into the periplastidial compartment PPC and across all four membranes into the stroma [142].

**Figure 6:**
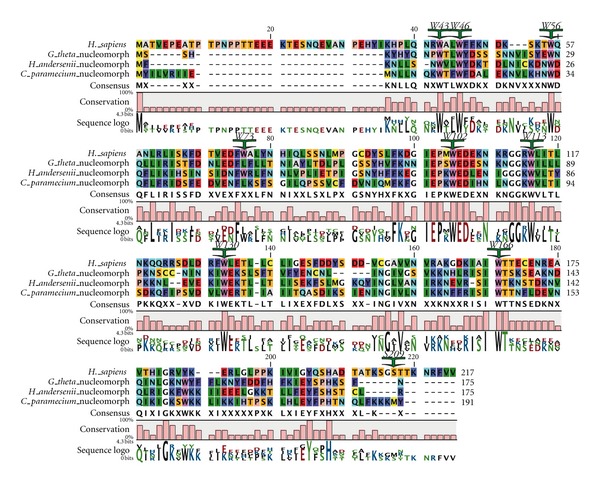
Comparison of the sequences of selected nucleomorph eIF4Es. Alignment of the amino acid sequences of the eIF4E from the nucleomorphs of *Guillardia theta*, *Haplogloia andersonii,* and *Cryptomonas paramecium*. Amino acid sequences were aligned with T-coffee using the BLOSUM62MT scoring matrix in CLC Main Workbench. To facilitate comparison of the sequences, the residues conserved in Class I eIF4Es in multicellular organisms are indicated and numbered as in human eIF4E-1: W43, W46, W56, W73, W102, W113, W130, W166, and S209.

The predicted eIF4E sequence of the *G. theta* nucleomorph is compacted, lacking extended amino-terminal and carboxy-terminal domains relative to the core of prototypical eIF4E (Figure 6) [6]. Although comparable forms from yeast, produced from deletion mutants, are still able to support life, they show considerably slower growth rates [143, 144]. This is likely to reflect a role of the N-terminal domain in enhancing stability. Scrutiny of the alignment also shows that the nucleomorph eIF4E has Leu at amino acid positions equivalent to V69 and W73 in human eIF4E-1. In human eIF4E-1, it is known that mutation to give a nonaromatic amino acid at position W73 disrupts the interaction with the adaptor protein, eIF4G, as does mutation of V69 to G [56, 57]. It is therefore unclear whether the nucleomorph eIF4E has the capacity to bind to eIF4G or indeed whether it needs to. It is possible that the nucleomorph eIF4E interacts with eIF4G imported from the host cytoplasm, although the sequence of the eIF4G-binding domain makes this unlikely. Alternatively, mRNA recruitment via an alternate interaction may be occurring. Interestingly, eIF4E sequences are available from additional nucleomorphs, those of another cryptophyte *Cryptomonas paramecium *and the heterokont *Haplogloia andersonii*. Both of these are truncated at the N-terminus, and both show substitutions in essential amino acids in the eIF4G binding domain.

## 13. Entamoeba and Mimivirus

Mimivirus is a double-stranded DNA virus isolated from amoebae [145]. It was first isolated from the water of a cooling tower in Bradford, England, during a study following a pneumonia outbreak in 1992 [146, 147]. Its name is derived from “mimicking microbe” because of the bacterium-like appearance of the particle and its Gram^+^ staining. It has a cycle of viral transmission and replication that is typical of many dsDNA viruses. The study of mimivirus grown in *Acanthamoeba polyphaga* reveals a mature particle with the characteristic morphology of an icosahedral capsid with a diameter of at least 400 nm. At the beginning of the life cycle, the virus enters the amoeba and the viral genome is released. After expression of viral proteins and replication of the genome, the virus DNA is packaged into capsids and viral particles are released from the amoeba [148]. Mimivirus has the largest known viral genome, 1.18 megabase pairs, and predicted to contain 1,262 genes, a very complex life cycle at the molecular level [146]. It encodes an unprecedented number of components of the transcriptional, translational, and replication machinery, many of which have not previously been described in viruses.

Although the mimivirus genome has more components resembling cellular genes than any other virus, it is still dependent on its host cell for the synthesis of proteins. Currently, the strategies by which mimivirus appropriates the host translation machinery have not been uncovered. Mimivirus exhibits many features that distinguish it from other nucleocytoplasmic large DNA viruses (NCLDVs). The most unexpected is the presence of numerous genes encoding central protein-translation components, encoding 10 proteins central to the translation apparatus: four aminoacy tRNA synthetases, eIF4E, ORF L496, eIF1A, eIF4A, eEF-1, and peptide chain release factor eRF1 [149, 150]. In addition, mimivirus encodes its own mRNA capping enzyme, and its own RNA cap guanine-N2 methyltransferase [151, 152]. Interestingly, mimivirus does not encode the mimic of the *α*-subunit of eIF2, found in many NCLDVs, that functions as a substrate to protect endogenous eIF2 from phosphorylation by an infection-activated kinase PKR. Finding these components of the translation apparatus in mimivirus calls into question the prevailing view that viruses rely entirely on the host translation machinery for protein synthesis [153]. Although the molecular mechanisms of its replicative cycle are yet to be uncovered, the detailed genome analysis has provided useful information on what viral genes may be involved in DNA replication and DNA repair, transcription, and protein folding, virion morphogenesis, and intracellular transport and suggests a complex life cycle.

**Figure 7:**
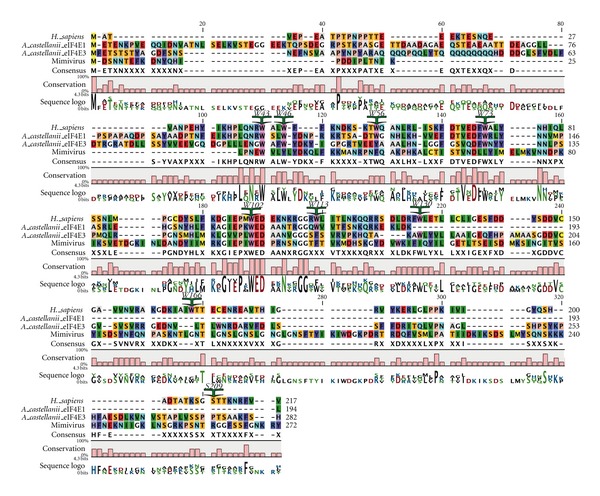
Comparison of the sequence of mimivirus eIF4E with those of an *Acanthamoeba* species. Alignment of the amino acid sequences of mimivirus eIF4E with eIF4Es from its host *Acanthamoeba castellani*. Amino acid sequences were aligned with T-coffee using the BLOSUM62MT scoring matrix in CLC Main Workbench. *A. castellani* sequences were derived from the Protist EST Program (PEP) in advance of scientific publication and acceptance by GenBank at http://amoebidia.bcm.umontreal.ca/public/pepdb/agrm.php. This site is no longer publically available. To facilitate comparison of the sequences, the residues conserved in Class I eIF4Es in multicellular organisms are indicated and numbered as in human eIF4E-1: W43, W46, W56, W73, W102, W113, W130, W166, and S209.

The atypical eIF4E-family member of mimivirus is shown in Figure 7 aligned with the amino acid sequences of eIF4E-family members from *Acanthamoeba*. Mimivirus eIF4E has F49, W109, and E110, in positions equivalent to W56, W102, and E103 of human eIF4E-1, predicting that it should function in cap binding. However, mimivirus eIF4E, like the many protist eIF4E-family members, has extended stretches of amino acids between structural units of the core tryptophans. The positions of these stretches in mimivirus eIF4E resemble the extensions found in eIF4E-family members from Alveolata and Stramenopiles. However, the stretch of amino acids between residues equivalent to W102 and W166 of mouse/human eIF4E-1 are considerably longer in mimivirus eIF4E than those found in *P. falciparum* or other known stramenopile/alveolate eIF4E family members. Mimivirus eIF4E also differs from other eIF4E-family members in that it lacks a Trp residue equivalent to W73 of mouse eIF4E-1 suggesting that the protein may not interact with eIF4G or 4E-BPs.

The host *A. castellanii* expresses at least five eIF4E-family members. None of the *A. castellanii* eIF4E family members shows extended stretches of amino acids in positions similar to those found in mimivirus eIF4E. Furthermore, *A. castellanii* eIF4E-family members possess conserved residues equivalent to V69 and W73 of human eIF4E-1 important for interaction with eIF4G and 4E-BPs, unlike mimivirus eIF4E. As a consequence of these differences in significant residues, it seems unlikely that mimivirus eIF4E has been acquired from the *Acanthamoeba* host. The sequence of mimivirus eIF4E predicts that it is likely to bind to 5′-cap structures but may not interact with eIF4G, suggesting that it could function as an inhibitor of cap-dependent translation. However, the mimivirus genome encodes genes for mRNA capping enzymes [151, 152], as do related NCLD viruses, suggesting that mimivirus mRNAs are capped and that the virus requires cap-dependent translation of its mRNAs. Since mimivirus can use both *A. polyphaga* and human as hosts, it will be of use to consider the role of its eIF4E in the context of mRNA recruitment in both environments.

## 14. Overview of Protist eIF4Es

Like multicellular eukaryotes, many protists encode multiple eIF4E family members. However, these do not fall into the eIF4E classes found in plants/metazoans/fungi. Of the eight conserved tryptophan residues typical of eIF4E Class I sequences, most are either conserved in protist eIF4E family members or are replaced by other aromatic residues. In many bikont protists, extensions are found between the conserved aromatic amino acids which vary with clade and phylogenetic grouping.  Figure 2 shows the relationships of the protist eIF4Es and suggests that they fall into three clades. eIF4Es from dinoflagellates/*Perkinsus* and heterokonts can be found in all three clades. eIF4Es from ciliates and the parasitic dinoflagellate *Amoebophrya* are present in only two clades. Unfortunately, at the current time, there are many more eIF4E sequences available for alveolates and excavates than for other protist groups, particularly the opisthokonts and amoebozoa. Furthermore, there is insufficient data on the functional characteristics of the eIF4Es in each of these clades to allow for any confident classification at this stage. Nevertheless, it is known that the *Leishmania* and *Trypanosoma* eIF4Es, EIF4E3 and 4 function as initiation factors and that the dinoflagellate eIF4E-2s from *K. veneficum* bind cap structures suggesting that this clade contains eIF4E family members that function as initiation factors. Table 2 shows the characteristics of some of the members from “Clades 1” and “2.” As genome sequencing projects are completed, it is expected that the number of protist eIF4E family members available for scrutiny will increase dramatically in the near future. A wider representation of taxa will allow a more complete understanding of the relationships between these eIF4Es, as will a much needed expansion of functional studies particularly in the non-parasitic representatives.

## Figures and Tables

**Table 1:** Eukaryotic groups and genera used for analysis of eIF4E family members. The six hypothesized supergroups of eukaryotes after Parfrey et al. [8]. Groups (pink) and genera (yellow) from which eIF4E sequences have been used to examine the relationship of protist eIF4E family members are highlighted.

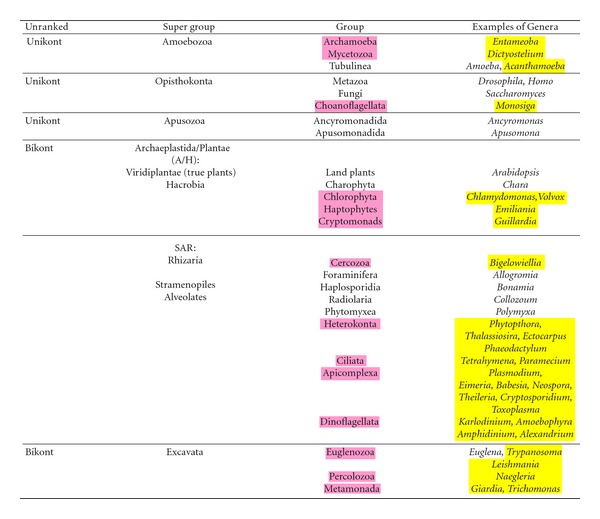

**Table 2:** Summary of protist eIF4E family member characteristics. A selection of Clade 1 and Clade 2 protist eIF4E family members is shown, looking at the residue at positions equivalent to W46, W56, W73, W113 in human eIF4E-1; presence or absence of a Ser residue at the position equivalent to S209 in human eIF4E-1; presence or absence of insertions; the sequence of the sequence of the eIF4G-binding domain. Shading indicates Amoebozoa (Pale yellow); nucleomorph (Gray); haptophyte (Aqua); alveolate, apicomplexan (Yellow); alveolate dinoflagellate/Perkinsus (Pink); excavate (Aquamarine) eIF4Es.